# Splenic Architecture and Function Requires Tight Control of Transmembrane TNF Expression

**DOI:** 10.3390/ijms23042229

**Published:** 2022-02-17

**Authors:** Kim C. M. Jeucken, Merlijn H. Kaaij, Jasper Rip, Charlotte C. N. van Rooijen, Yik Y. Kan, Odilia B. J. Corneth, Jan Piet van Hamburg, Sander W. Tas

**Affiliations:** 1 Department of Experimental Immunology, Amsterdam University Medical Centers, University of Amsterdam, 1105 AZ Amsterdam, The Netherlands; k.c.jeucken@amsterdamumc.nl (K.C.M.J.); m.h.kaaij@amsterdamumc.nl (M.H.K.); c.c.n.vanrooijen@amsterdamumc.nl (C.C.N.v.R.); y.y.kan@amsterdamumc.nl (Y.Y.K.); j.p.vanhamburg@amsterdamumc.nl (J.P.v.H.); 2 Department of Clinical Immunology and Rheumatology, Amsterdam Rheumatology and Immunology Center, Amsterdam University Medical Centers, University of Amsterdam, 1105 AZ Amsterdam, The Netherlands; 3 Department of Pulmonary Medicine, Erasmus MC, University Medical Center Rotterdam, 3015 GD Rotterdam, The Netherlands; j.rip@erasmusmc.nl (J.R.); o.corneth@erasmusmc.nl (O.B.J.C.); 4 Department of Immunology, Erasmus MC, University Medical Center Rotterdam, 3015 GD Rotterdam, The Netherlands

**Keywords:** transmembrane TNF, spleen, lymph node, B cell, T cell, follicle, TNFR, germinal center, IgA, plasma cell

## Abstract

Soluble tumor necrosis factor (sTNF) is an important inflammatory mediator and essential for secondary lymphoid organ (SLO) development and function. However, the role of its transmembrane counterpart (tmTNF) in these processes is less well established. Here, the effects of tmTNF overxpression on SLO architecture and function were investigated using tmTNF-transgenic (tmTNF-tg) mice. tmTNF overexpression resulted in enlarged peripheral lymph nodes (PLNs) and spleen, accompanied by an increase in small splenic lymphoid follicles, with less well-defined primary B cell follicles and T cell zones. In tmTNF-tg mice, the spleen, but not PLNs, contained reduced germinal center (GC) B cell fractions, with low Ki67 expression and reduced dark zone characteristics. In line with this, smaller fractions of T follicular helper (Tfh) and T follicular regulatory (Tfr) cells were observed with a decreased Tfh:Tfr ratio. Moreover, plasma cell (PC) formation in the spleen of tmTNF-tg mice decreased and skewed towards IgA and IgM expression. Genetic deletion of TNFRI or –II resulted in a normalization of follicle morphology in the spleen of tmTNF-tg mice, but GC B cell and PC fractions remained abnormal. These findings demonstrate that tightly regulated tmTNF is important for proper SLO development and function, and that aberrations induced by tmTNF overexpression are site-specific and mediated via TNFRI and/or TNFRII signaling.

## 1. Introduction

Secondary lymphoid organs (SLOs), including spleen and lymph nodes (LNs), are central sites for the initiation of adaptive immune responses. To efficiently establish these responses, SLOs have a tightly organized architecture enabling efficient interactions between incoming (naive) immune cells and local antigens, presenting as immune and stromal cells [1]. In general, this architecture includes T and B cell zones, the latter containing germinal centers (GCs) where high affinity, long-lived PCs, and memory B cells are generated [2].

TNF can be expressed in either a soluble TNF (sTNF) or a transmembrane TNF (tmTNF) form. The release of sTNF is dependent on tmTNF cleavage by ADAM17 (a disintegrin and metalloproteinase 17, or TNF converting enzyme (TACE)). sTNF signals via TNFRI (p55, expressed across all cells) and TNFRII (p75, expressed on specific cell types, including myeloid cells and stromal cells) [3,4,5]. In contrast to sTNF, tmTNF has a dual role as it not only mediates signaling via TNFRI and II ligation, but can also function as a receptor itself via reverse signaling upon cell-cell contact with TNFRI and TNFRII expressing cells [5].

The cytokine TNF is intimately involved in the organization of SLO architecture. TNF is produced by a variety of cell types—predominantly macrophages, but also lymphocytes and stromal cells [3,4]. Although SLOs can develop in TNF-deficient (TNF^−/−^) mice, defects in the spatial organization of LNs and spleen have been described, including aberrant morphology of B cell follicles, GCs, and follicular dendritic cell (FDC) networks [6,7]. The observed defects seem mainly dependent on TNFRI signaling, as mice lacking TNFRI have an abnormal SLO morphology, which is comparable to TNF^−/−^ mice. This morphology includes aberrant architecture of lymphoid follicles, as mice lacking TNFRI have a peripheral B cell sheet rather than individual lymphoid follicles [7,8]. We recently demonstrated that PLNs (brachial and inguinal) of TNFRII^−/−^ mice have a morphology reminiscent of TNFRI^−/−^ mice, since PLNs of these mice have decreased individual lymphoid follicles, suggesting a previously unknown role for TNFRII signaling in SLO architecture [8]. 

In line with the well-established contribution of TNF-TNFR signaling to SLO architecture organization, there is evidence for a specific role for tmTNF in this process. Via experimental models that express physiological levels of tmTNF, but not sTNF, it has been shown that tmTNF can support general SLO structure in the absence of sTNF. However, SLOs in these mice had defects in primary B cell follicle formation, indicating an essential role for sTNF in this process that cannot be replaced by tmTNF [9,10]. 

An important drawback of murine models that lack sTNF but express physiological levels of tmTNF is that it is difficult to identify processes that solely depend on tmTNF and are not shared with sTNF. Since deletion of tmTNF automatically also leads to loss of sTNF there are no gene targeting approaches available to delete tmTNF while maintaining physiological levels of sTNF. Therefore, it is extremely difficult to perform studies to identify specific roles of tmTNF. 

One alternative strategy to study tmTNF-specific functions is via tmTNF overexpression rather than inhibition or deletion. In this study, we used an experimental mouse model (Tg86) that has enforced tmTNF expression in the presence of physiological sTNF levels due to introduction of a mutant TNF gene construct harboring a defect in the ADAM17 cleavage site (muTNF_Δ1–12_). This generates an increase in tmTNF expression while retaining physiological sTNF levels [11,12]. 

In this study, we investigated the effects of tmTNF overexpression on SLO architecture and lymphoid follicle associated lymphocyte populations. In addition, we explored the contribution of TNFRI and –II signaling to the observed changes. 

## 2. Results

### 2.1. tmTNF Overexpression Leads to Enlarged SLOs and Aberrant Splenic Lymphoid Follicle Structures

**Figure 1:**
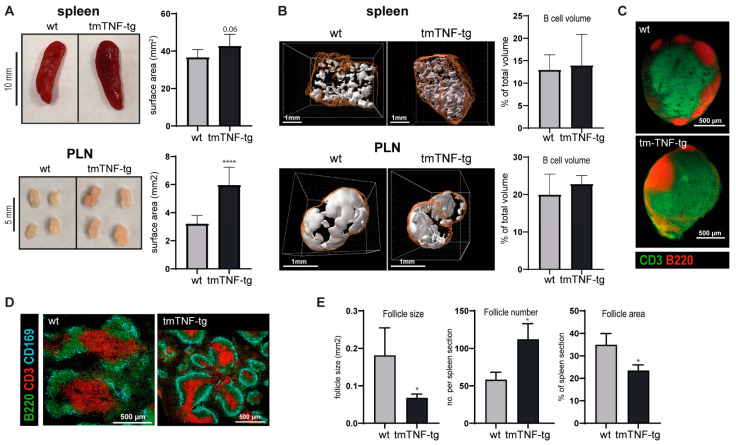
Spleen and lymph nodes of tmTNF-tg mice have enlarged SLOs and abnormal splenic lymphoid follicle structures. Spleen and PLNs were histologically analyzed. (**A**) Representative pictures of the spleen and PLNs of tmTNF-tg and wt mice. SLOs of tmTNF-tg mice are macroscopically enlarged compared to wt mice (*n* = 3 PLN; *n* = 5 spleen). (**B**) Representative three-dimensional reconstructions of whole PLN and spleen sections. Ultramicroscopy analysis revealed no changes in B cell volume of tmTNF-tg SLOs compared to wt (*n* = 3; white = B cell areas, orange = tissue outline). (**C**) Immunofluorescent staining indicated that PLNs of tmTNF-tg mice develop normal B cell follicles, representative images. (**D**) Representative image of a spleen of a tmTNF-tg mouse. Splenic lymphoid follicles contained B and T cell areas with defined CD169^+^ marginal zones (CD3 = marker for T cells; B220 = marker for B cells). (**E**) Spleen of 17-week-old tmTNF-tg mice have a decrease in lymphoid follicles size and follicle area (as percentage of the total surface of the spleen), but an increase in follicle number (*n* = 6). (* *p* ≤ 0.05, **** *p* ≤ 0.0001).

Mice overexpressing tmTNF develop clinical symptoms resembling spondyloarthritis (SpA) [11]. A prominent feature of an immune response is the activation and enlargement of SLOs, which can be observed for example as a result of immunization [13]. Consistent with the clinical features and inflammation in tmTNF-tg mice, we found that PLNs, but not the spleen of these mice, were macroscopically enlarged compared to their wt littermates (Figure 1A). As a next step, we analyzed the effects of tmTNF overexpression on lymphoid follicle architecture. Using ultramicroscopy imaging, we performed whole tissue 3-dimensional reconstruction of PLN and spleen to visualize and analyze B220^+^ cell distribution in these tissues. These analyses showed that B220^+^ follicles were present in the spleen and PLN of tmTNF-tg mice (Figure 1B). Moreover, similar ratios of B220^+^ volume of the total volume of spleen and PLN tissue were observed in tmTNF-tg and wt mice. In the spleen of tmTNF-tg mice, but not in PLN, the density of the B220^+^ follicles visually appeared to increase, whereas the individual follicle size appeared to be decreased (Figure 1B). PLNs of tmTNF-tg mice also had a normal distribution of CD3^+^ T cells (Figure 1C). To examine the splenic lymphoid follicle phenotype in tmTNF-tg mice in more detail, we performed a staining for CD3 and B220 in combination with CD169, a marker for marginal zone macrophages [14]. The lymphoid follicles in the spleen of tmTNF-tg mice contained both B and T cell areas, as well as clearly defined CD169^+^ marginal zones (Figure 1D and Figure S1A). Quantification of the lymphoid follicles in HE stained sections revealed that the number of individual follicles was significantly increased in the spleen of tmTNF-tg mice, while their average size significantly decreased (Figure 1E). These differences were already detectable in tmTNF-tg mice of 5 weeks and became more apparent over time (Figure S1B).

Taken together, these histological analyses demonstrate that although lymphoid follicles with defined B and T cell areas develop in SLOs of tmTNF mice, the morphology of the spleen was abnormal with an increase in the number of lymphoid follicles, which were smaller in size in comparison to the follicles in the spleen of wt mice.

### 2.2. tmTNF Overexpression Reduces GC B Cell Formation in the Spleen but Not in PLN

**Figure 2:**
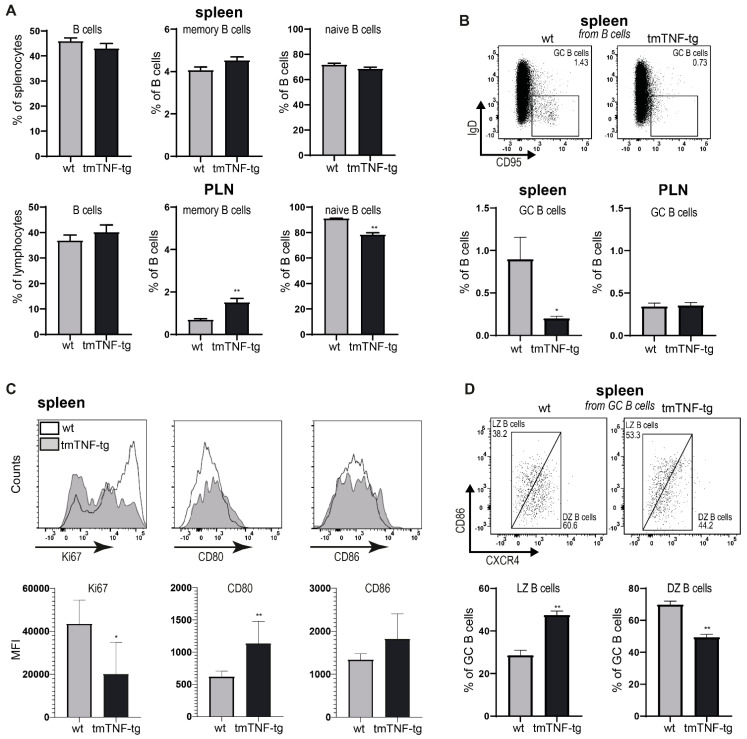
Reduced GC B cell formation in the spleen of tmTNF-tg mice. Different B cell populations were analyzed by flow cytometry. (**A**) Spleen and PLNs of tmTNF-tg mice have normal total B cell proportions (left panels), an increase in memory B cells (middle panels) and a decrease in naïve B cells (right panels). (**B**) Representative dot plots showing the gating strategy to separate CD95^+^IgD^−^ GC B cells (from B220^+^CD19^+^ populations). The spleen of tmTNF-tg mice had decreased proportions of GC B cells compared to wt mice. No changes were found in GC B cell proportions in PLNs. (**C**) Representative histograms of Ki67, CD80, and CD86 (left panels). In the GC B cells from the spleen of tmTNF-tg mice, Ki67 expression decreased, and CD80 and CD86 expression levels increased. (**D**) Representative dot plots showing the gating strategy to separate CD86^+^CXCR4^−^ LZ GC B cells and CD86^−^CXCR4^+^ DZ GC B cells (from B220^+^CD19^+^CD95^+^IgD^−^ populations). In the spleen of tmTNF-tg mice, LZ B cell proportions increased and DZ B cell proportions decreased compared to wt. GC = germinal center; LZ = light zone; DZ = dark zone; MFI = mean fluorescent intensity; * = *p* ≤ 0.05; ** = *p* ≤ 0.01; *n* = 6 for all graphs.

The organizational aberrancies in the spleen of tmTNF-tg mice prompted us to investigate the B cell compartment in the SLOs of tmTNF-g mice in more detail. In line with the ultramicroscopy-derived data, similar proportions of CD19^+^B220^+^ B cells were found in the spleen and PLNs of tmTNF-tg mice in comparison to wt mice. The PLNs, but not spleen of tmTNF-tg mice showed an increased proportion of memory B cells (CD19^+^B220^+^CD80^+^PD-L2^+^), which was accompanied by a decrease in naïve B cells (CD19^+^B220^+^IgD^+^) (Figure 2A).

In line with the smaller size of the B cell follicles in the spleen, a significant reduction in the proportion of B220^+^IgD^−^CD95^+^ GC B cells was present in the spleen of tmTNF-tg mice, whereas the GC B cell fraction was unaffected in PLNs (Figure 2B). Upon examining the GC B fraction in the spleen of tmTNF-tg mice in more detail, we found that the proliferative capacity of these cells was significantly reduced as indicated by decreased expression of the proliferation marker Ki-67. In contrast, expression levels of the costimulatory markers CD80 and CD86 showed a similar (CD86) or even increased (CD80) activation status of GC B cells from tmTNF-tg mice (Figure 2C). Similar findings for Ki-67, CD80, and CD86 were observed in GC B cells from PLNs of tmTNF-tg mice (Figure S2A). GC B cells can be further divided in light zone (LZ) and dark zone (DZ) B cells by the differential expression of CD86 and CXCR4. Using this strategy, we found that the spleen of tmTNF-tg mice had an increase in LZ B cells and a decrease in DZ B cells (Figure 2D). This observation was in line with the observed decrease in Ki-67 expression, as the DZ is the site of GC proliferation. These effects of tmTNF overexpression on LZ and DZ B cell characteristics were similar to our previous findings in the ectopic lymphoid structures (ELS) present in the vertebrae of tmTNF-tg mice [15]. In contrast to the spleen, PLN LZ B cells decreased while DZ B cells increased in tmTNF-tg mice (Figure S2B), which is consistent with the systemic inflammation observed in these mice. 

These data show that the aberrancies in the architecture of the spleen in tmTNF-tg mice were accompanied with changes in the B cell compartment, including a reduced proportion of GC B cells. These cells also showed a reduced proliferative capacity without losing their capacity for CD80/86 co-activation.

### 2.3. tmTNF Overexpression Results in Increased IgA and IgM Switching 

**Figure 3:**
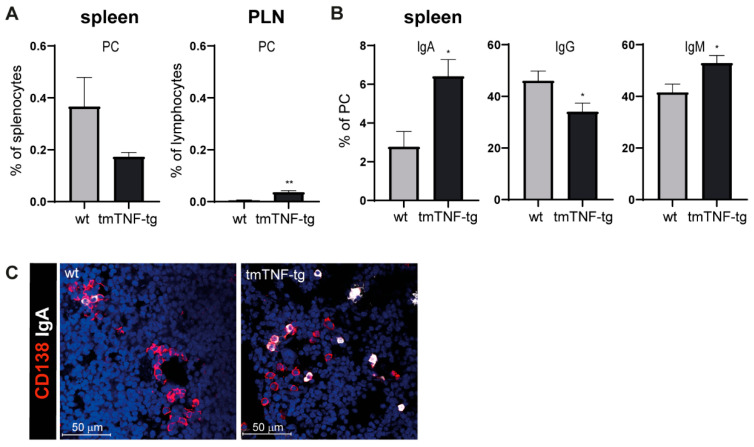
Increase in IgA and IgM plasma cell proportions in the spleen of tmTNF-tg mice. PC populations were analyzed using flow cytometry and immunofluorescent staining. (**A**) PC proportions in the spleen and PLNs of tmTNF-tg mice. (**B**) Analysis of PC Ig-classes in spleen samples. IgA^+^ and IgM^+^ PC populations were increased in the spleen of tmTNF-tg mice. (**C**) Representative image of an immunofluorescent staining of spleen sections. IgA^+^ PC were increased in the spleen of tmTNF-tg mice. PC = plasma cell; Ig = immunoglobulin; CD138^+^ = marker for PC; * = *p* ≤ 0.05; ** = *p* ≤ 0.01; *n* = 6 for all graphs.

As a next step the PC compartment in PLNs and spleen of wt and tmTNF-tg mice was investigated. A significant increase was found in the proportion of PCs in PLNs of tmTNF-tg mice. In sharp contrast, a trend for a lower proportion of PCs was observed in the spleen of tmTNF-tg mice, albeit not significantly (Figure 3A). The analysis of the Ig class specificity of PCs in the spleen of tmTNF-tg mice showed that the proportion of IgA and IgM class-switched PCs was significantly increased, at the expense of IgG^+^ PC formation (Figure 3B). The presence of increased ratios of IgA^+^ PCs was confirmed by IF stainings of CD138 and IgA on spleen sections of tmTNF-tg and wt mice (Figure 3C). 

Collectively, overexpression of tmTNF results in an increase in PC cells in PLNs but not in the spleen. Remarkably, tmTNF overexpression resulted in skewed class-switching in the spleen in favor of IgA and IgM.

### 2.4. tmTNF Overexpression Affects GC-Associated T cells in Spleen

**Figure 4:**
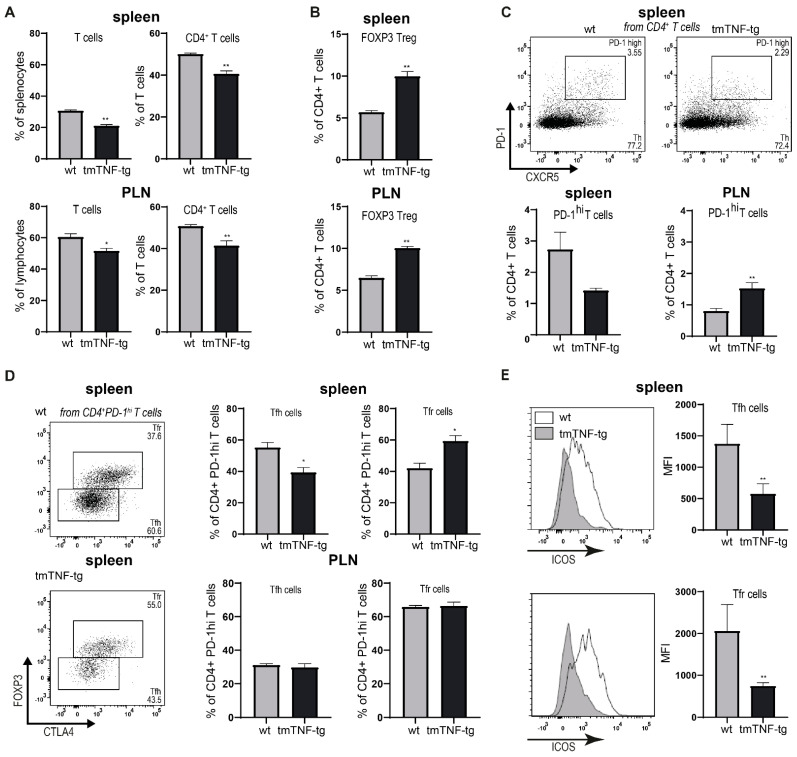
The spleen of tmTNF-tg mice has aberrant proportions of germinal center-associated T cell populations. Different GC T cell populations were analyzed by flow cytometry. (**A**) Decrease in total T cell and CD4^+^ fractions in spleen and PLN of tmTNF-tg mice. (**B**) Increase in the fraction of FOXp3 Treg cells in the spleen and PLN of tmTNF-tg mice. (**C**) Representative dot plots showing the gating strategy to separate PD-1^high^ T cells (from CD3^+^CD4^+^ populations). PD-1^high^ T cell proportions were decreased in the spleen and increased in PLNs of tmTNF-tg mice. (**D**) Representative dot plots showing the gating strategy to separate FOXP3^+^CTLA4^+^ Tfr cells from FOXP3^−^CTLA4^−^ Tfr cells (from PD-1^high^ populations). (**E**) The Tfh cell proportion decreased and Tfr cell proportion increased in the spleen of tmTNF-tg mice. No changes were found in PLN of tmTNF-tg mice compared to wt mice. GC = germinal center; Treg = regulatory T cell; Tfr = T follicular regulatory cells; Tfh = T follicular helper cell; * = *p* ≤ 0.05; ** = *p* ≤ 0.01; *n* = 6 for all graphs.

Next, the effect of tmTNF overexpression on the T cell compartment was examined. This analysis showed that the proportion of total CD3^+^ T cells, as well as the CD3^+^CD4^+^ T cell fraction was reduced in both the spleen and PLNs of tmTNF-tg mice (Figure 4A). Within the CD4^+^ T cell compartment there was a strong increase in FOXP3^+^ T regulatory cells (Tregs) in both the spleen and PLNs (Figure 4B). Within SLOs, the specialized follicular CD4^+^ T cell subsets Tfh and Tfr cells are important in orchestrating and regulating GC reactions. Tfh cells are essential for the formation of GCs and the development of high affinity antibody-producing B cells [16]. In contrast, Tfr cells are important for restraining Tfh cell-mediated functions and thereby controlling antibody responses [17]. Flow cytometric analysis revealed that the spleen of tmTNF-tg mice had a significant decrease in follicular-associated (CXCR5^+^PD1^+^) CD4^+^ T cells. In sharp contrast, this follicular CD4^+^ T population was strongly induced in PLNs of tmTNF-tg mice (Figure 4C). A closer examination of this follicular population revealed that in the spleen of tmTNF mice the Tfh population was reduced while the Tfr population was induced. In contrast, there were no effects of tmTNF overexpression on the proportions of Tfh and Tfr cells in PLNs (Figure 4D). 

As a measure of potential B cell costimulatory properties, we measured ICOS [18] expression and found that both Tfh and Tfr cells in the spleen of tmTNF-tg mice had a strong reduction of ICOS expression compared to wt mice (Figure 4E). 

Together, these data show that the reduced proportion of GC B cells in the spleen of tmTNF-tg mice is accompanied by lower numbers of follicular CD4^+^ T cells, a reduced Tfh/Tfr ratio, and a potential loss of B cell costimulatory properties of Tfh.

### 2.5. The Effects of tmTNF Overexpression on Chemokine and Adhesion Molecule Expression in the Spleen 

**Figure 5:**
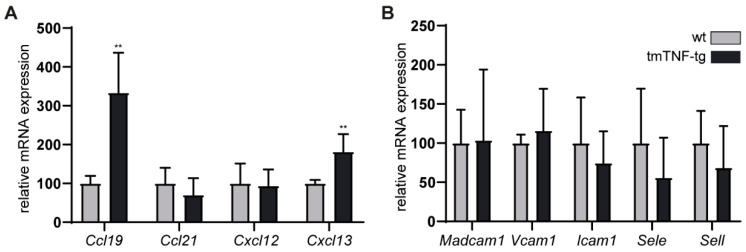
Expression of GC chemokines and adhesion molecules in the spleen of tmTNF-tg mice. mRNA levels of different chemokines and adhesion molecules were analyzed in spleen of tmTNF-tg mice. (**A**) mRNA expression of different GC-associated chemokines. tmTNF-tg spleen had an increase in *Ccl19* and *Cxcl13*. (**B**) No change in mRNA expression of different GC-associated adhesion molecules in tmTNF-tg mice. GC = germinal center; ** = *p* ≤ 0.01; *n* = 6.

Chemokines and adhesion molecules play an essential role in recruiting and restraining lymphocytes to lymphoid follicles and GC formation. To analyze the possible contribution of these molecules to the observed alterations in the spleen of tmTNF-tg mice, mRNA levels of key GC-associated chemokines and adhesion molecules were determined. For the chemokines (CCL19/CCL21; migration to T cell zone, CXCL13; DZ, and CXCL12; LZ), we found normal (*Ccl21*, *Cxcl12*) to significantly increased (*Ccl19*, *Cxcl13*) transcription levels compared to the spleen of wt mice (Figure 5A). Since adhesion molecules are important in maintaining lymphocytes within the GC once they arrive, we evaluated mRNA levels of adhesion molecules (MadCAM1, VCAM1, ICAM1I, SELE, SELL) within the spleen. Similar transcription levels were found between the spleens of tmTNF-tg and wt mice (Figure 5B). The normal or even higher transcription levels of chemokines and adhesion molecules suggests that the changes within GCs of the spleen of tmTNF-tg mice are not due to the absence or lower levels of these chemoattractants.

### 2.6. Splenic Defects Caused by tmTNF Overexpression Are Mediated via TNFRI and TNFRII Signaling

**Figure 6:**
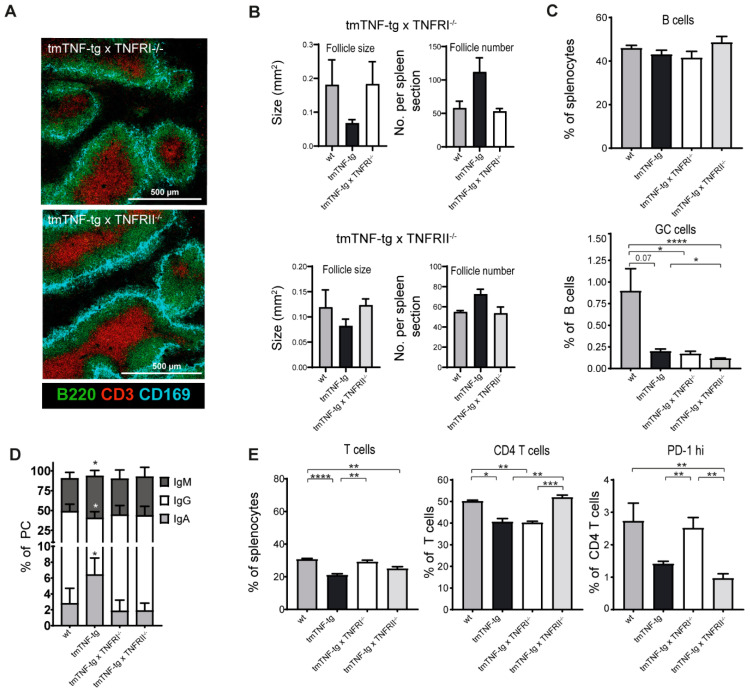
Effects of TNFRI and TNFRII deficiency on tmTNF-induced effects in lymphoid follicles of the spleen. Different spleen populations were analyzed via flow cytometry and immunofluorescent staining. (**A**) Representative images of spleens obtained from tmTNF-tg × TNFRI^−/−^ and tmTNF-tg × TNFRII^−/−^ mice. Splenic lymphoid follicles contained B and T cell areas with defined CD169^+^ marginal zones (CD3 = marker for T cells; B220 = marker for B cells). (**B**) Restoration of follicle size and number in the spleen of tmTNF-tg × TNFRI^−/−^ mice (left panels) and tmTNF-tg × TNFRII^−/−^ (right panels) to levels comparable to wt mice (*n* = 2–3). (**C**) Analysis of B cell and GC B cell numbers. Total B cell proportions were not changed in the spleen of tmTNF-tg × TNFRI^−/−^ and tmTNF-tg × TNFRII^−/−^ mice. GC B cell proportions were comparable in the spleen of tmTNF-tg and tmTNF-tg × TNFRI^−/−^ mice and further decreased in spleen of tmTNF-tg × TNFRI^−/−^ mice compared to the spleen of tmTNF-tg mice. TNFRI or –II knock-out in tmTNF mice did not affect GC LZ and DZ ratios (*n* = 6). (**D**) Analysis of PC Ig-classes in spleen samples. IgA^+^ and PC populations were decreased in tmTNF-tg × TNFRI^−/−^ and tmTNF-tg × TNFRII^−/−^ mice compared to tmTNF-tg mice and restored to levels comparable to wt mice. Test for significance for wt mice in comparison to the individual transgenic mouse lines (*n* = 6). (**E**) Analysis of GC-associated T cells. The T cell fraction increased in tmTNF-tg × TNFRI^−/−^ mice compared to tmTNF-tg mice and restored to levels comparable to wt mice. The CD4^+^ T cell proportion increased in tmTNF-tg × TNFRII^−/−^ mice compared to tmTNF-tg mice and restored to levels comparable to wt mice. PD-1^hi^ T cells were increased in tmTNF-tg × TNFRI^−/−^ mice compared to tmTNF-tg mice and restored to levels comparable to wt mice and further decreased in tmTNF-tg × TNFRII^−/−^ mice (*n* = 6). GC = germinal center; LZ = light zone; DZ = dark zone; PC = plasma cell; Ig = immunoglobulin; * = *p* ≤ 0.05; ** = *p* ≤ 0.01; *** = *p* ≤ 0.001; **** = *p* ≤ 0.0001).

tmTNF is capable of inducing signaling events via TNFRI and TNFRII [5]. By crossing tmTNF-tg mice to either a TNFRI or a TNFRII deficient background, the contribution of TNFRI and TNFRII signaling on the splenic tmTNF-tg phenotype was investigated. CD3/B220/CD169 stainings showed the presence of lymphoid follicles with defined B and T cells areas that are clearly aligned with CD169^+^ marginal zone macrophages (Figure 6A and Figure S3A). 

The size and number of the lymphoid follicles appeared to be normal in tmTNF-tg mice on both the TNFRI^−/−^ and TNFRII^−/−^ background (Figure 6B). However, flow cytometric analysis revealed that tmTNF overexpression on both TNFRI^−/−^ and TNFRII^−/−^ backgrounds still resulted in reduced GC B proportions as compared to wt mice (Figure 6C). In contrast, TNFRI or TNFRII deficiency overcame tmTNF-induced IgA skewed class switching (Figure 6D). In the T cell compartment, the inhibitory effect of tmTNF overexpression on the total T cell fraction was restored both on the TNFRI^−/−^ and TNFRII^−/−^ backgrounds. Furthermore, TNFRII, but not TNFRI deficiency, restored the tmTNF-induced reduction in CD4^+^ T cells to normal levels. Intriguingly, the fraction of follicular PD1^+^ CD4^+^ T cells was restored to normal levels in tmTNF-tg mice on the TNFRI^−/−^ background, whereas this was not the case and even reduced in tmTNF-tg mice on the TNFRII^−/−^ background (Figure 6E). The Tfh:Tfr ratio was not restored to normal levels in tmTNF-tg mice on both the TNFRI^−/−^ and TNFRII^−/−^ backgrounds (Figure S3B). 

These data show that the effects of tmTNF overexpression on follicle number, size, and IgA class switching in the spleen is mediated by both TNFRI and TNFRII signaling. Deficiency of TNFRI or TNFII alone is however not sufficient to restore the fraction of GC B cells in the presence of tmTNF overexpression. Although both TNFRI and TNFRII deficiency were able to restore the total fraction of T cells, TNFRI and TNFRII are differentially involved in the tmTNF-induced alterations of CD4^+^ T cell fractions.

## 3. Discussion

Although the essential role of sTNF in SLO development, architecture, and function is evident from multiple studies [6,19,20,21,22], the role of tmTNF in these processes is less established. Here, we show that tmTNF overexpression has clear effects on spleen morphology and splenic lymphoid follicle development. Overexpression of tmTNF resulted in an increased number of lymphoid follicles in the spleen, which were on average reduced in size. In addition, these lymphoid follicles contained reduced fractions of GC B and follicular T cells, and PCs displayed skewed class switching in favor of IgA^+^ and IgM^+^ PCs. 

The tmTNF overexpression approach that we used for this study enabled us to investigate the effects of increased tmTNF expression in the presence of physiological levels of sTNF [12]. The tmTNF transgene is under control of the TNF promoter, which makes tmTNF overexpression limited to cells prone to producing TNF [12]. This situation may mimic pathological conditions, as observed in patients with autoimmune diseases, including SpA, who have increased tmTNF levels in the presence of sTNF [11]. Our data also adds to studies in which the role of tmTNF has been investigated in the absence of sTNF [10] or to studies that used gene targeting approaches or inhibitors against ADAM17 to prevent TNF secretion [23,24,25]. For the latter, it is difficult to distill TNF-specific effects as ADAM17 is involved in the cleavage of a wide range of membrane proteins [23].

Mice deficient in TNF lack the formation of clear individual primary B cell follicles in the spleen; they rather have a peripheral B cell ring-like structure that surrounds a T cell zone [10,20,21]. In the spleen of tmTNF-tg mice, individual B cell follicles are present and can be mainly observed in the more developed and larger lymphoid follicles. The larger and more developed splenic lymphoid follicles in the spleen of tmTNF-tg mice show a clear segregation of T and B cell zones. However, they display a rather irregular shape more in line with the lymphoid follicles observed in the spleen of mice that express tmTNF in the absence of sTNF [10]. In the absence of sTNF, tmTNF is capable of inducing lymphoid follicles surrounded by marginal zone macrophages [10,20]. Here, we show that overexpression of tmTNF results in the formation of new lymphoid follicles over time, which are surrounded by a CD169 marginal zone. However, this de novo induction results in development of less mature follicles, which lack primary B cell follicles and distinct B and T cell areas. This suggests that increased levels of tmTNF in the presence of physiological levels of sTNF can induce lymphoid follicles, but that their development into fully mature follicles, consisting of primary B cells follicles, GC B cells, and follicular T cells is impaired. It might be possible that the abnormal tmTNF:sTNF ratio in tmTNF-tg mice [12] prevents the full maturation of splenic follicles. 

The development of splenic lymphoid follicles and GC formation is central to humoral responses that are induced under inflammatory conditions and in experimental autoimmune disease models such as collagen induced arthritis (CIA) [13,26,27]. tmTNF-tg mice display clear signs of (systemic) inflammation and clinical features of SpA. In addition, they exhibit vertebral ELS formation [11,15]. Therefore, it is surprising that the spleen of these mice lacks mature splenic follicles and that even reduced proportions of GC B cells and follicular T cells are present. Our data suggests a suppressive role for tmTNF in the full maturation of splenic follicles. This notion is supported by a study of CII-immunization in TNF deficient mice, which reported the development of splenomegaly, increased splenic memory CD4^+^ T cells, but not B cells, and impaired IgG class switching in comparison to wt CII-immunized mice [13]. This may be in line with the finding that TNF also has an immunomodulatory role by regulating Treg cell responses, i.e., supporting stability, expansion, function, and IL-2 production [28,29]. In this context, it is interesting to note that the Treg fraction increased in the spleen of tmTNF-tg mice, while the CD4^+^ T cell fraction as a whole decreased. Moreover, the Tfr fraction in the spleen increased in disfavor of Tfh cells. Since Tfr cells are regulators of Tfh cells and thereby GC B cell activation and GC reactions [16,17,18], it is tempting to hypothesize that the immunomodulatory role of tmTNF in supporting Treg and Tfr cell expansion and function may underlie a lack of full maturation of splenic lymphoid follicles in tmTNF-tg mice. Since ICOS plays an important role in GC B cell co-stimulation and Tfh migration [16,30,31], the reduced ICOS expression on Tfh and Tfr cells in tmTNF-tg mice could play a role in the reduced proliferation rate and number of GC B cells and/or lower proportions of follicular Tfh cells that were observed in the spleen of these mice. It is striking that the effects of tmTNF overexpression were evident on splenic follicles, while, except from a reduced proliferation rate of GC B cells, no clear effects were observed (other than those expected during systemic inflammation) on PLN lymphoid structures, GC B cells, and follicular T cell fractions. It may be possible that different mechanisms are at play in spleen and PLN, and that the role of tmTNF is different in specific SLOs. This idea is supported by the finding that the cellular TNF source (soluble or transmembrane) varies between different SLOs [32]. In this context, the specific deletion of TNF in T cells, in contrast to the deletion of TNF in B cells, has no obvious effect on the formation of splenic primary B cell follicles. In PLN, TNF is expressed by both B and T cells, whereas in the spleen TNF is mainly produced by B cells, which may explain this phenomenon [32]. Moreover, TNFRI deficient mice fail to develop GCs in the spleen after immunization but do develop clear PNA positive GCs in PLN [33]. This indicates that the signals required for formation of lymphoid follicles and B cell activation differ between the spleen and PLNs. Moreover, in our study we found that the Tfh:Tfr ratio decreased in the spleen, but not in the PLNs of tmTNF-tg mice. This aberrancy may also underlie the specific effects of tmTNF on lymphoid follicle maturation observed in the spleen but not in PLNs.

Overexpression of tmTNF resulted in a skewed class, switching in favor of IgA and IgM at the expense of the percentage of IgG^+^ PCs, which decreased. This is in line with the increase in systemic IgA levels that we found earlier in tmTNF-tg mice [15]. It may be possible that due to the reduced proportion of GC B cells, there is a reduction of the IgG^+^ PC output in the spleen. The splenic lymphoid follicles still had an intact marginal zone. Since IgA^+^ and IgM^+^ PCs are mainly derived from the marginal zone B cells [34,35], this could be an explanation as to why IgA^+^ and IgM^+^ PCs are still produced in splenic follicles of tmTNF-tg mice. On the other hand, tmTNF may also exert a direct effect on Ig class switching. Interestingly, TNF can induce activation-induced cytidine deaminase (AID), an essential factor for class switching, via NF-κB signaling [36]. Moreover, non-canonical NF-κB which can be activated via TNFRs has been implicated in the production of IgA [37,38].

Chemokines and adhesion molecules play an essential role in the homing and activation of lymphocytes at specific SLO locations [39]. Earlier findings showed that tmTNF in the absence of sTNF is capable of inducing chemokine expression in the spleen to relatively normal levels [10]. It may, therefore, be possible that the decrease in splenic GC B cells and follicular T cells in the context of tmTNF overexpression is related to aberrant expression of chemokines and adhesion molecules. However, we found normal levels for most of the examined key chemokines and cytokines but observed an increase in expression of the genes encoding for CXCL13 and CCL19. These chemokines are recognized by CXCR5 expressing GC B cells and follicular T cells, or by CCR7 expressing naïve T cells, respectively [27,39]. The increased levels of CXL13 make it an unlikely cause for the decreased splenic fractions of GC B cells and follicular T cells in tmTNF-tg mice. Nevertheless, a dysregulation or altered lymphocyte homing and activation of lymphocytes cannot be formally ruled out on the basis of these data as it is a complicated process involving many additional molecules in addition to the ones analyzed here.

The lack of TNFRI or TNFRII signaling in tmTNF-tg mice restored the size and number of splenic follicles to normal, indicating that aberrant lymphoid follicle formation via tmTNF depends on TNFRI or TNFRII. In contrast, the deficiency of TNFRI or TNFRII was not sufficient to restore splenic GC B cell populations and IgA-skewed class switching in tmTNF-tg mice. 

In the absence of TNFRI or TNFRII, T cell proportions in the spleen of tmTNF-tg mice (partly) returned to normal. The CD4^+^ T cell fraction in the spleen of tmTNF-tg mice normalized on a TNFRII deficient background, but not on TNFRI deficient background. This may be related to the role of TNFRII signaling in Treg expansion and function [28,40]. However, the Tfh:Tfr ratio was not affected in tmTNF-tg mice on both the TNFRI- and TNFRII-deficient background.

These data imply that tmTNF signals, via both TNFRI and TNFRII, and redundancy or cross-talk [41] may be present, explaining why the absence of either one of these receptors is insufficient in preventing the effects of tmTNF overexpression. It could also be that the additional defects inherent to the lack of TNFRI or TNFRII signaling play a role. In both TNFRI^−/−^ and TNFRII^−/−^, mice follicles in the spleen are able to develop, though TNFRI^−/−^ mice show aberrancies in the formation of primary B cell follicles, marginal zones, and follicular dendritic cells. In addition, TNFRI^−/−^ mice do not respond adequately on immunization as GC formation and T cell dependent IgG production was affected [42]. In contrast, TNFRII^−/−^ mice do not show obvious effects on splenic organization and function [42,43].

Although tmTNF can signal via either TNFRI or TNFRII, preferential binding of tmTNF to these receptors may play a role. Some reports argue for preferential binding of sTNF to TNFRI, thereby serving as the mediator of the pro-inflammatory effects of TNF. In contrast, tmTNF may preferentially bind to TNFRII [28,44,45]. A hint for the underlying mechanisms of tmTNF overexpression induced splenic aberration may be derived from the expression pattern of TNFRI and TNFRII. Whereas TNFRI is ubiquitously expressed, TNFRII expression is mainly limited to specific immune cells, including Tregs and B regulatory cells. Consequently, TNFRII has been implicated to mediate homeostatic and regulatory effects, rather than inducing inflammation [40,45,46,47,48,49].

Uncontrolled TNF-TNFR signaling is associated with the pathogenesis of many diseases, including immune-mediated inflammatory disorders (IMIDs) such as SpA, rheumatoid arthritis, and inflammatory bowel disease [50]. Originally, the pathogenic effects of TNF were mainly attributed to sTNF, but there is accumulating evidence that tmTNF can also contribute to pathology [5]. For example, tmTNF expression in the gut is predictive for anti-TNF treatment response in Crohn’s disease [51]. Moreover, we recently found that the overexpression of aberrant tmTNF expression in mice results in SpA features, including pathological bone formation, arthritis, ELS formation in the bone marrow of vertebrae, and increased serum IgA levels [11,15]. Further dissection of the role of tmTNF in these SpA features, together with the observed effects on SLOs and lymphoid cells, would be instrumental in advancing our understanding of the role of tmTNF in SpA and potentially other IMIDs. This knowledge could subsequently be exploited to optimize anti-inflammatory therapies targeting TNF.

The findings highlighted in this report illustrate that tmTNF exerts context dependent functions and that tight regulation of tmTNF expression levels and signaling via TNFRI and TNFRII is critical for the architecture and function of the spleen. In the context of tmTNF overexpression, follicles are formed in the spleen, but their structural appearance and cellular composition is altered, which may be caused by effects on GC B cells or T cell mediated regulatory actions. 

## 4. Materials and Methods

### 4.1. Mice

tmTNF-tg mice (TgA86), provided by Prof. G. Kollias, have a murine TNF_Δ1-12_-human ß-globin hybrid gene construct containing the murine TNF-a gene promoter and were on the C57Bl/6 background [11,12]. Heterozygous tmTNF-tg and wild-type (wt) littermates were used for the experiments. tmTNF-tg × TNFRI^−/−^ and TNFRII^−/−^ animals were generated by crossing tmTNF-tg mice with TNFRI^−/−^ (Tnfrsf1a^tm1Imx^) and TNFRII^−/−^ (Tnfrsf1b^tm1Mwm^) strains (Jackson Laboratory, Bar Harbor, ME, USA). All animals were genotyped using PCR [11,12]. Animals were bred and housed in specific pathogen-free conditions in the animal facility of Amsterdam University Medical Centers (AUMC), location Academic Medical Center (AMC). Breeding, housing, and experiments were approved by the ethical committee of animal welfare of the AUMC and the University of Amsterdam in accordance with the central authority for scientific procedures on animals (CCD). 

### 4.2. Sample Processing

Spleens and peripheral LNs (PLNs; brachial and inguinal) from mice of various ages were collected in cold PBS. Excess tissue was removed using fine forceps to keep the tissue intact. For histological analyses, organs were embedded in Tissue Tek O.C.T. compound (Sakura, Torrance, CA, USA) and placed at −80 °C and cut into 5 µm thick sections with CM1950 Leica cryostat (specimen and chamber set at −17 °C). Samples intended for whole mount tissue immunolabeling were fixed in methanol-free PFA (Thermo Fisher Scientific, Waltham, MA, USA) for 8 h and washed overnight with PBS at 4 °C and saved in 0.02% NaN_3_/PBS until further processing.

### 4.3. Flow Cytometry

Single-cell suspensions of spleen and PLNs were made by passing the tissue through a 100 μm cell mesh filter in PBS supplemented with 0.5% BSA and 2 mM EDTA). Next, 2 × 10^6^ cells were incubated with antibody cocktails for intra- and extracellular markers and various controls [15]. Intracellular immunoglobulin staining was done by fixation and permeabilization of cells with BD Cytofix/Perm Buffer and BD Perm/Wash buffer (BD Biosciences, Franklin Lakes, NJ, USA), respectively. For intracellular staining, cytokine-expressing cells were fixed in 2% paraformaldehyde (PFA)/PBS and permeabilized and stained in 0.5% saponin (Sigma-Aldrich, Darmstadt, Germany). Labeling of FOXP3 was done using the Foxp3 staining kit (eBioscience, San Diego, CA, USA), according to manufacturers’ instructions. Measurements were performed on LSRII flow cytometer (BD Biosciences) and results were analyzed using FlowJo software (v10.6, TreeStar Inc., Ashland, OR, USA).

### 4.4. Histology 

Basic histological analysis was done using hematoxylin and eosin (HE) staining. Here, 40 µm sections were stained with hematoxylin solution (Mayer’s, Sigma-Aldrich) (8 min), washed with running tap water (10 min), briefly dipped in distilled water and incubated with 96% ethanol (30 sec) before counterstaining with eosin solution (Sigma-Aldrich) (3 min). Next, sections were dehydrated in ethanol and xylene series (5 min 96% and 2× 5 min 100% ethanol; 2× 5 min xylene). Sections were covered with xylene based mounting medium (Entellan, Merck, Darmstadt, Germany) and air dried. Images were acquired with DM6 and analysis of lymphoid follicle surface was done using ImageJ (v1.53k, National Institutes of Health, Bethesda, MD, USA).

### 4.5. Immunofluorescence Staining

Here, 5-20 µm sections were fixed with acetone (10 min, room temperature (RT)), permeabilized with 0.2% Triton X-100/PBS (10 min, RT), and blocked with 0.5% BSA/PBS (30 min, RT). Next, sections were incubated with primary antibodies; B220-Alexa Flour 488 (AF488), CD3-AF594, CD169-AF647, and CD138 (Biolegend, San Diego, CA, USA), and IgA-AF647 (Southern Biotech, Birmingham, AL, USA), in 0.5% BSA. Conjugated antibodies were incubated for 1h at RT in dark and unlabeled CD138 was incubated overnight 4 °C in dark, followed by incubation with AF594-conjugated donkey-anti-rat Ig (Thermo Fisher Scientific) in 0.5% BSA (30 min, RT, dark). Sections were covered with ProLong Antifade or Fluoromount-G (Invitrogen, Waltham, MA, USA). Nuclear staining was done with DAPI (Prolong antifade, Thermo Fisher Scientific) or by supplementing secondary antibody-cocktails with Hoechst (1:1000; Invitrogen). Images were acquired with a Leica TCS SP8 X mounted on a Leica DMI6000 inverted microscope and analyzed with Las X software (Leica Biosystems, Nussloch, Germany). Whole mount tissue immunolabeling and 3D imaging was performed as described earlier [8].

### 4.6. RNA Isolation and Quantitative RT-PCR

RNA was isolated using the GenElute^TM^ Mammalian Total RNA Miniprep kit (Sigma-Aldrich) according to manufacturer’s instructions. cDNA was synthesized using a first strand cDNA synthesis kit (Thermo fisher Scientific). Quantative RT-PCR was done via SybrGreen assay (Applied Biosystems) with an input of 5 ng cDNA and the following primers (forward, reverse): *Ccl19*: cctgggaacatcgtgaaagc, tagtgtggtgaacacaacagc; *Ccl21*: atgtgcaaaccctgaggaag, ttgagggctgtgtctgttca; *Cxcl12*: tgcatcagtgacggtaaacca, cacagtttggagtgttgaggat; *Cxcl13*: caacgctgcttctcctcct, ggcgtaacttgaatccgatct; *Madcam1*: cctggccctagtaccctacc, tctgtacactgcccaagctg; *Vcam1*: cccgtcattgaggatattgg, ggtcattgtcacagcaccac; *Icam1*: ttcacactgaatgccagctc, gtctgctgagacccctcttg; *Sele*: atgaagccagtgcatactgtc, cggtgaatgtttcagattggagt; *Sell*: tacattgcccaaaagcccttat, cctccttggacttcttgttgtt; *B2m* (reference gene): catggctcgctcggtgacc, aatgtgaggcgggtggaactg. Analysis was done using StepOne Plus (Applied Biosystems, San Francisco, CA, USA).

### 4.7. Statistics

Data of 2 groups were analyzed with the Mann–Whitney U test and multiple group comparisons were done using the Kruskal–Wallis test. All tests were calculated using GraphPad Prism v8.2.1 (Graphpad Software Inc., San Diego, CA, USA). For all analyses differences were considered significant for *p*-values ≤ 0.05 (* *p* ≤ 0.05, ** *p* ≤ 0.01, *** *p* ≤ 0.001, **** *p* ≤ 0.0001). Data is shown as mean and SEM.

## Data Availability

Not applicable.

## Supplementary Materials

The following supporting information can be downloaded at https://www.mdpi.com/article/10.3390/ijms23042229/s1.
