# Supplementary material for: Splenic Architecture and Function Requires Tight Control of Transmembrane TNF Expression
Source: Int J Mol Sci. 2022 Feb 17;23(4):2229. doi: 10.3390/ijms23042229 (PMC8876982; doi:10.3390/ijms23042229)
Supplement: Supplementary file 1 [file ijms-23-02229-s001.zip › Supplementary Figure 1.pdf]

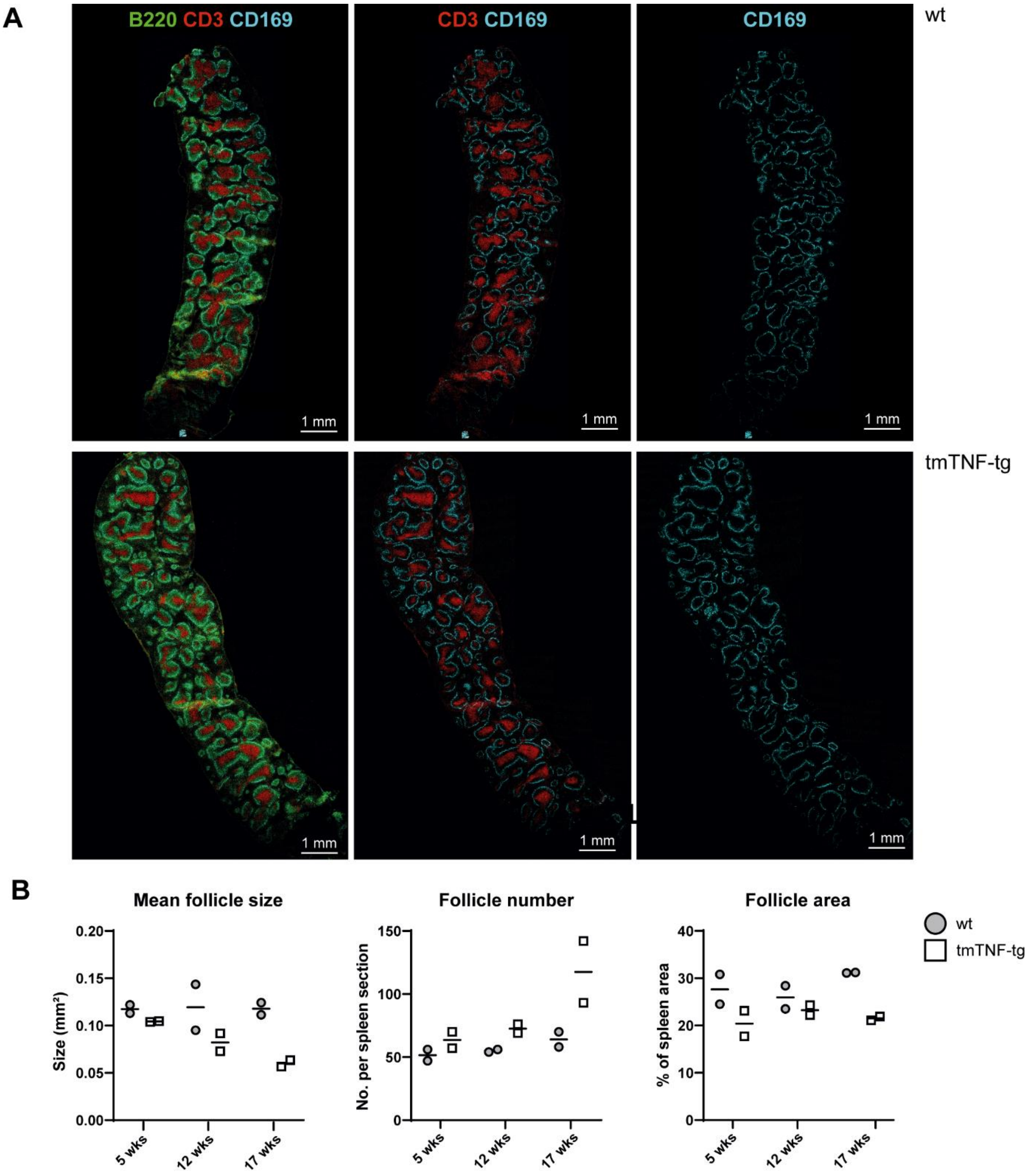

Figure S1. Splenic follicles in tmTNF-tg spleen. (**a**) Representative overview images of tmTNF-tg and wt spleen. (**b**) Follicle size, number and area in tmTNF-tg and wt mice of different ages.
