# Supplementary material for: Splenic Architecture and Function Requires Tight Control of Transmembrane TNF Expression
Source: Int J Mol Sci. 2022 Feb 17;23(4):2229. doi: 10.3390/ijms23042229 (PMC8876982; doi:10.3390/ijms23042229)
Supplement: Supplementary file 1 [file ijms-23-02229-s001.zip › Supplementary Figure 2.pdf]

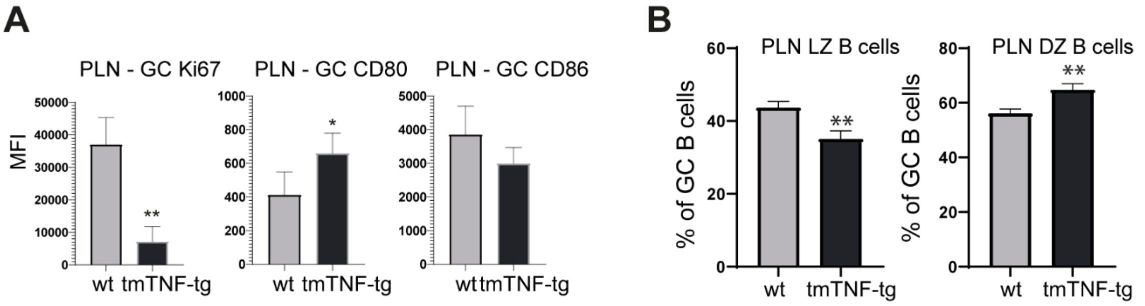

Figure S2. Germinal center B cells in tmTNF-tg PLN. (**a**) Expression of Ki67, CD80 and CD86 in PLN of tmTNF-tg mice. (**b**) GC LZ and DZ numbers in tmTNF-tg PLN.
