# Supplementary material for: Splenic Architecture and Function Requires Tight Control of Transmembrane TNF Expression
Source: Int J Mol Sci. 2022 Feb 17;23(4):2229. doi: 10.3390/ijms23042229 (PMC8876982; doi:10.3390/ijms23042229)
Supplement: Supplementary file 1 [file ijms-23-02229-s001.zip › Supplementary Figure 3.pdf]

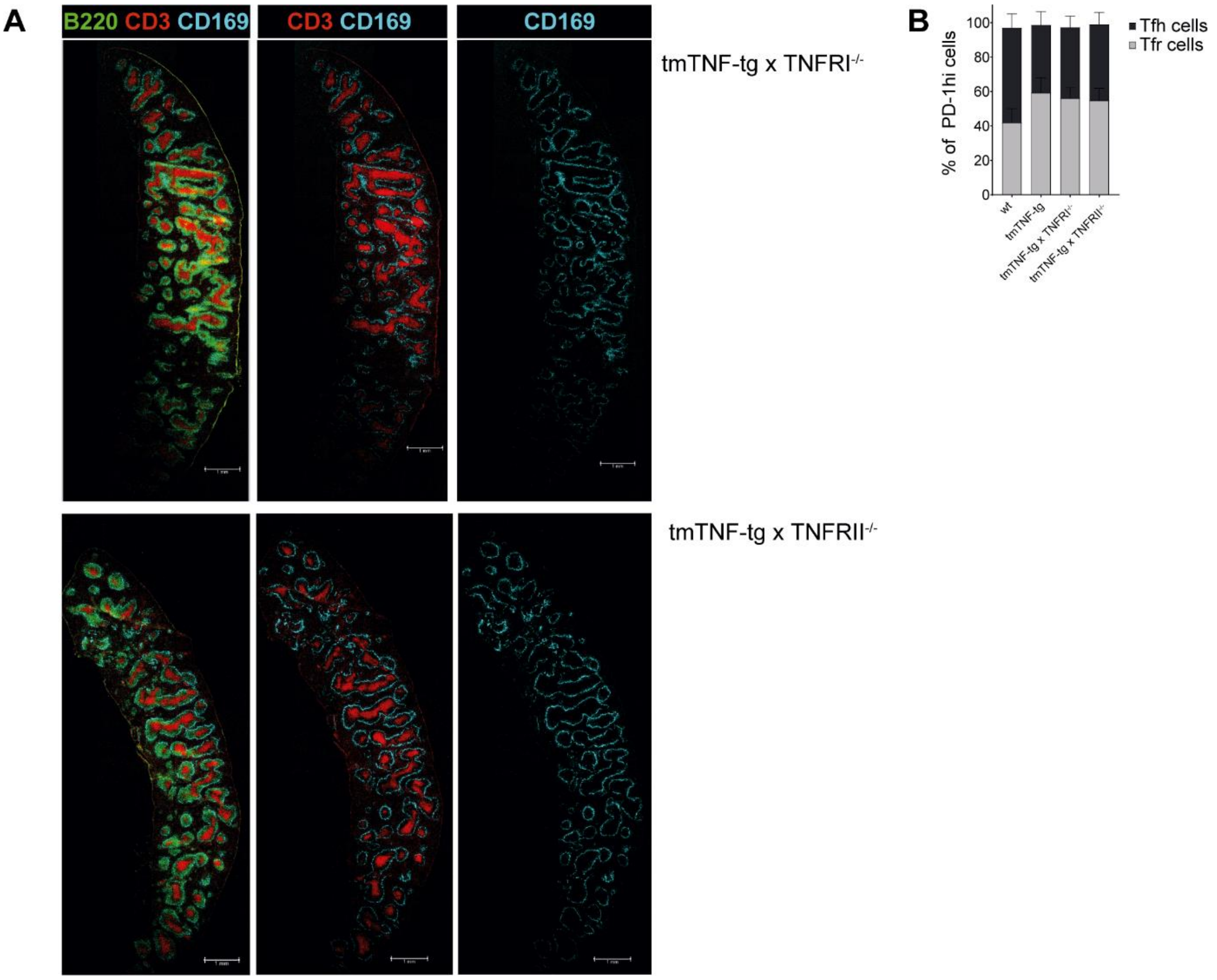

Figure S3. Splenic follicles in tmTNF-tg x TNFRI$^{-/-}$ and tmTNF-tg x TNFRI$^{-/-}$ spleen. (**a**) Representative overview images of tmTNF-tg x TNFRI$^{-/-}$ and tmTNF-tg x TNFRI$^{-/-}$ spleen.
